# Microbial Community Response to Simulated Petroleum Seepage in Caspian Sea Sediments

**DOI:** 10.3389/fmicb.2017.00764

**Published:** 2017-04-28

**Authors:** Marion H. Stagars, Sonakshi Mishra, Tina Treude, Rudolf Amann, Katrin Knittel

**Affiliations:** ^1^Department of Molecular Ecology, Max Planck Institute for Marine MicrobiologyBremen, Germany; ^2^Department of Marine Biogeochemistry, GEOMAR – Helmholtz Centre for Ocean Research KielKiel, Germany; ^3^Department of Earth, Planetary and Space Sciences, University of California, Los Angeles, Los AngelesCA, USA; ^4^Department of Atmospheric and Oceanic Sciences, University of California, Los Angeles, Los AngelesCA, USA

**Keywords:** hydrocarbon degradation, alkyl succinate synthase, MasD, sulfate reduction, microbial diversity, crude oil

## Abstract

Anaerobic microbial hydrocarbon degradation is a major biogeochemical process at marine seeps. Here we studied the response of the microbial community to petroleum seepage simulated for 190 days in a sediment core from the Caspian Sea using a sediment-oil-flow-through (SOFT) system. Untreated (without simulated petroleum seepage) and SOFT sediment microbial communities shared 43% bacterial genus-level 16S rRNA-based operational taxonomic units (OTU_0.945_) but shared only 23% archaeal OTU_0.945_. The community differed significantly between sediment layers. The detection of fourfold higher deltaproteobacterial cell numbers in SOFT than in untreated sediment at depths characterized by highest sulfate reduction rates and strongest decrease of gaseous and mid-chain alkane concentrations indicated a specific response of hydrocarbon-degrading Deltaproteobacteria. Based on an increase in specific CARD-FISH cell numbers, we suggest the following groups of sulfate-reducing bacteria to be likely responsible for the observed decrease in aliphatic and aromatic hydrocarbon concentration in SOFT sediments: clade SCA1 for propane and butane degradation, clade LCA2 for mid- to long-chain alkane degradation, clade Cyhx for cycloalkanes, pentane and hexane degradation, and relatives of *Desulfobacula* for toluene degradation. Highest numbers of archaea of the genus *Methanosarcina* were found in the methanogenic zone of the SOFT core where we detected preferential degradation of long-chain hydrocarbons. Sequencing of *masD*, a marker gene for alkane degradation encoding (1-methylalkyl)succinate synthase, revealed a low diversity in SOFT sediment with two abundant species-level MasD OTU_0.96_.

## Introduction

Petroleum mainly consists of aliphatic hydrocarbons (alkanes), naphthenes, aromatics, asphaltenes, and other compounds in varying composition depending on where and how it was formed. A large and diverse number of microorganisms, including bacteria, archaea, and fungi, have evolved the ability to utilize these hydrocarbons as sources of food and energy for growth under either oxic or anoxic conditions (Grossi et al., 2008; Heider and Schühle, 2013).

**FIGURE 1:**
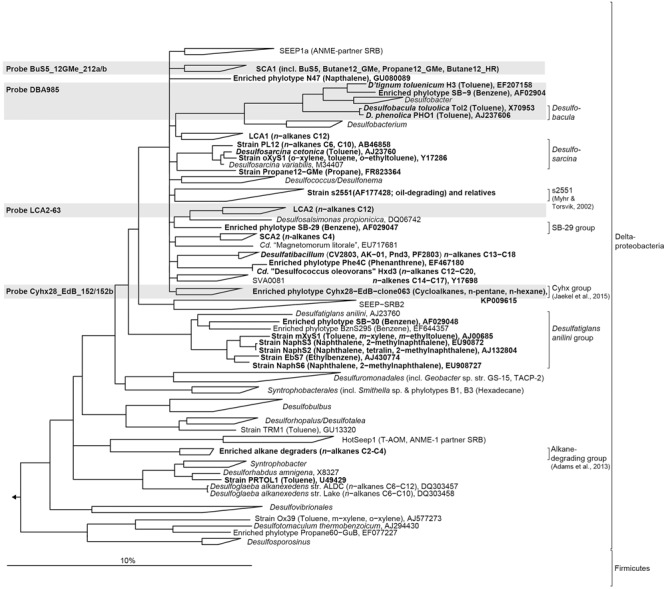
**Phylogenetic tree showing the affiliation of 16S rRNA sequences of cultured or enriched sulfate-reducing anaerobic hydrocarbon degraders to selected reference sequences of the domain Bacteria.** Substrate usage of hydrocarbon degraders is given in parentheses. Hydrocarbon-degrading clades or species of which relatives were detected in the 16S rRNA gene sequence dataset from sediment-oil-flow-through (SOFT) core sediment (based on genus-level; >94.5% sequence similarity) have been printed in boldface type. The gray boxes show coverage of probes used for CARD-FISH. The bar represents 10% estimated sequence divergence.

Contaminations of an ecosystem with hydrocarbons as observed, e.g., after the *Deepwater Horizon* disaster in the Gulf of Mexico (Atlas and Hazen, 2011; Kimes et al., 2013, 2014), have important consequences on the autochthonous microbial communities, which suffer drastic changes in structure and function. In the oxic water column the majority of oil is degraded by aerobic microbes, while oil from “natural spills” at hydrocarbon seeps is mainly degraded by anaerobic microbes living in benthic environments (Head et al., 2003; Jones et al., 2008). Using sulfate, nitrate, manganese, or ferric iron as electron acceptors anaerobic enrichment cultures were established from marine seep sediments, oil reservoirs and petroleum polluted sites (e.g., Kniemeyer et al., 2007; Mbadinga et al., 2012; Acosta-González et al., 2013; Jaekel et al., 2013; Bian et al., 2015) and several isolates have been obtained (**Figure 1**). Furthermore, hydrocarbon-degrading syntrophic enrichment cultures have been established under methanogenic conditions (e.g., Zengler et al., 1999; Chang et al., 2006; Berdugo-Clavijo and Gieg, 2014; Embree et al., 2014). Responsible strains can degrade only a narrow range of hydrocarbon sources and belong to the phyla Proteobacteria and Firmicutes within the domain Bacteria or to Euryarchaeota within the domain Archaea. A common way of anaerobic non-methane alkane activation is its addition across the double bond of fumarate to form alkyl-substituted succinates, a step catalyzed by a glycyl radical enzyme, 1-methyl alkyl succinate synthase [Mas, Grundmann et al., 2008; Rabus et al., 2016; also known as alkylsuccinate synthase (Ass, Callaghan et al., 2008)]. Other known mechanisms for non-methane alkane activation include anaerobic hydroxylation followed by carboxylation and the oxygen-independent hydroxylation (reviewed in Callaghan, 2013). Most cultivated anaerobic alkane degraders activate alkanes via fumarate addition. Thus, the gene encoding the catalytic subunit of the responsible enzyme Mas (*masD*) serves as relevant genetic marker and its study allows a cultivation-independent survey of the diversity and distribution of alkane-degrading communities in any anoxic hydrocarbon-impacted environment.

The fate of oil in the marine environment is subjected to microbial activity, thus altering the oil’s composition. The rate of microbial degradation depends on several environmental factors like nutrients, salinity, and availability of terminal electron acceptors, composition of petroleum, temperature and pressure (Atlas, 1981; Leahy and Colwell, 1990). Only few studies investigated anaerobic hydrocarbon degradation under close-to-in situ conditions and most of these studies have focused either on one or few selected hydrocarbons (Mbadinga et al., 2011; Jaekel et al., 2013, 2015; Johnson et al., 2015; Musat, 2015). Thus, the aim of this study was to follow the response of the benthic microbial community to simulated petroleum seepage in a marine sediment core. The Caspian Sea was selected as study site because it is one of the oldest petroleum-producing regions in the world with huge oil and gas reserves (Effimoff, 2000). Anthropogenic reasons such as offshore drilling, oil refineries and pipeline constructions as well as natural reasons such as hydrocarbon release from mud volcanoes (Akper, 2012) have led to pollution of Caspian Sea (Karpinsky, 1992; Dumont, 1995). Pollutants accumulate and are partly trapped, e.g., in surface sediments. So far only a few studies have studied petroleum-degrading microbial communities in sediments from the Caspian Sea (Hassanshahian et al., 2010, 2012; Hassanshahian, 2014; Mahmoudi et al., 2015).

In the accompanying study by Mishra et al. (this issue) a sediment-oil-flow-through (SOFT) system was established that simulated petroleum seepage-like conditions. In this SOFT system, whole round sediment cores from the Caspian Sea are supplied with petroleum flow from bottom to top (simulating a seep situation) and artificial oxic seawater from ventilated supernatant. Using these sediments from the SOFT experiment (Mishra et al., this issue) we here report the microbial 16S rRNA gene diversity after 190 days of simulated petroleum seepage. In comparison, untreated sediments were analyzed. Furthermore, the anaerobic alkane-degrading community was characterized based on *masD* gene diversity. We hypothesize that individual taxa respond specifically to simulated petroleum seepage by an increase of their cell numbers resulting in a change of community composition. We further hypothesize that this specific cell increase is vertically structured according to sequential petroleum-degradation. To address these hypotheses specific cell numbers of selected hydrocarbon-degrading taxa in SOFT and untreated sediments were determined by CARD-FISH, and molecular data obtained in this study were correlated with the biogeochemical and petroleum degradation data obtained by Mishra et al. (this issue).

## Materials and Methods

### Study Site and SOFT System

The study site Baku Bay is located in the SW Caspian Sea near Baku, Azerbaijan (N 39 59.548, E 49 28.775, Supplementary Figure S1). In the past, the Caspian Sea was, as remnant of the Paratethys Sea, connected to oceans, but has become landlocked about five million years ago. There is large influx of freshwater by numerous rivers that drain into the Caspian Sea, thus the salinity is only about one-third of that of seawater (1.3%, Millero and Chetirkin, 1980). In November 2012, several sediment cores (large cores: 30 cm long, 6 cm in diameter; small cores: 30 cm long, 2.6 cm in diameter) were collected from a coastal area at a water depth of ∼60 cm. Cores were directly sealed and stored at 10°C in the dark for 3 months until three large and two small cores were processed as reference (referred to as “untreated sediment”) upon arrival at GEOMAR, Germany. Cores were used for sediment pore water analyses (1 large core), sediment solid phase and molecular analyses (1 large core), and sulfate reduction activity (2 small cores; for details see Mishra et al., this issue). The third large core was stored at 0°C for further 2.5 months until the start of the SOFT system (referred to as “SOFT sediment”). After storage for 3 years at 0°C, another large sediment core (referred to as “untreated sediment-3 years”) was processed for bacterial community composition analysis. Although storage at 0°C, absence of light and the presence of anoxic conditions probably slowed down any kind of heterotrophic microbial activity and geochemical alterations, this core was used to track possible changes in diversity due to storage issues, in particular changes of oil-degrading deltaproteobacterial sulfate reducers.

Sediments were mostly sandy with porosity around 0.4. Measured concentrations of alkanes in untreated sediments were below the detection limit of <0.1 ng ml^-1^ (Mishra et al., this issue). Light (i.e., low viscosity/specific gravity), live crude oil (i.e., containing dissolved gas in solution) for the SOFT experiment was provided by Dea Deutsche Erdoel AG and originated from the North Sea, Mittelplate (sampled in February 2013). Seepage was simulated in the SOFT core by pumping petroleum at a constant rate through the core from bottom to top, while artificial oxic seawater diffused into the sediment from the ventilated supernatant. The experiment was stopped after incubation for 190 days at 16°C in the dark. Sediment cores (“untreated,” SOFT core, and “untreated-3 years”) were sliced in one or two centimeter thick slices and immediately frozen at -20°C until use for DNA extraction. Experimental set up of the SOFT core was too complicate for replication, yet we consider adjacent, neighboring layers as technical replicates. More details on sampling procedure, SOFT system setup, as well as pore water sulfate, sediment methane, and sulfate reduction activity measurements are provided in Mishra et al. (this issue).

### DNA Extraction

DNA was extracted from 1 g sediment per depth layer with the UltraClean soil DNA isolation kit (MO BIO, Carlsbad, CA, USA) according to the manufacturer’s protocol for maximum yields. An initial cell lysis step was introduced by adding 0.02 mg ml^-1^ proteinase K (Merck, Darmstadt, Germany) to the sample and incubating for 50 min at 37°C with moderate shaking. The sediments were then added to the bead solution tubes and the manufacturer’s protocol was followed.

### Sequencing of 16S rRNA Genes

Bacterial 16S rRNA genes were amplified from DNA extracted from (i) untreated sediments, (ii) untreated-3 years sediments, and (iii) SOFT sediments with the primer pair Bakt_341F (5′-CCTACGGGNGGCWGCAG-3′)/Bakt_785R (5′-GACTACHVGGGTATCTAATCC-3′) (Herlemann et al., 2011). For each sample, at least six replicate PCR reactions (20 μl volume) per primer pair were carried out containing each 0.5 μM primer each, 250 μM dNTPs, 0.3 μg/μl BSA, 1 × PCR buffer, 0.25 U Taq polymerase (5Prime, Germany) or alternatively 1 U Phusion High Fidelity Taq polymerase (New England Biolabs, Ipswich, MA, USA), and 10–25 ng template under the following conditions: initial denaturation at 95°C for 5 min, followed by 35 cycles of denaturation (96°C, 1 min), annealing (58°C, 1 min), elongation (72°C, 2 min), and a final elongation step (72°C, 10 min). The replicate PCR reactions were pooled, 500 bp-amplicons extracted from an agarose gel (1.5% w/v) and purified using the MinElute PCR Purification Kit (Qiagen) according to the manufacturer’s recommendations.

Amplicons retrieved from untreated and SOFT sediments were analyzed by massive parallel tag sequencing on a 454 Life Sciences GS FLX sequencer (Roche, Basel, Switzerland) at the Max Planck-Genome-Centre, Cologne, Germany. The raw reads were submitted to a rigorous quality control procedure using a mothur version 1.32.1 routine (Schloss et al., 2009) including trimming and quality filtering of the reads (quality scores ≥35 in a 50 nt window, no ambiguous bases, and homopolymers ≤ 8 nt). Chimeras were identified and removed using UCHIME (Edgar et al., 2011). Amplicons retrieved from untreated-3 years sediments were sequenced on a HiSeq 2500 sequencer (2x 250 bp; Illumina, San Diego, CA, USA) at the Max Planck-Genome-Centre, Cologne, Germany. Reads were quality trimmed (trimq = 20, minlength = 238) and merged (overlap >12) using software package BBmap v4.3 and split using mothur v1.33.3 (Schloss et al., 2009). Reads were further processed with a modified MiSeq SOP (Kozich et al., 2013, page accessed in May 2016) using mothur including uchime chimera removal (Edgar et al., 2011).

Archaeal 16S rRNA genes were amplified with primers Arch20F (5′-TTC CGG TTG ATC CYG CCR G-3′) (Massana et al., 1997)/Arch519R (5′-GGTDTTACCGCGGCKGCTG-3′; Sørensen and Teske, 2006). Primers were barcoded with the Ion Xpress barcodes and extended for ligation with Ion A and Ion truncated P1 adapters at the 5′ end. For each sample PCRs (50 μl volume) were carried out containing 0.5 μM primer each, 250 μM dNTPs, 0.3 μg/μl BSA, 1 × PCR buffer, 0.25 U Taq polymerase (5Prime, Germany), about 20 ng template under the following conditions: initial denaturation at 95°C for 5 min, followed by 30 cycles of denaturation (94°C, 1 min), annealing (58°C, 1 min), elongation (72°C, 3 min), and a final elongation step (72°C, 10 min). The reactions were separated on an E-Gel SizeSelect Gel (Invitrogen) and the 500 bp-amplicon extracted following the manufacturer’s protocol. Then, the PCR product was purified using the MinElute PCR Purification Kit (Qiagen) according to the manufacturer’s recommendations. Sequencing of an equimolar pool of different amplicons was carried out on the Ion Torrent Personal Genome Machine (PGM) system using the Ion PGM^TM^ Sequencing 400 Kit (both Life Technologies) following the standard protocol (Ion 314^TM^ Chip v2). The raw reads were submitted to a rigorous quality control procedure using BaseCaller V4.2 manufacturer’s default parameters including trimming and quality filtering of the reads with minor modifications (trim-qual-cutoff = 15; trim-qual-window-size = 20).

According to the revised taxonomic thresholds (Yarza et al., 2014) for a genus, which was updated to 94.5% sequence identity, the bacterial and archaeal quality reads were clustered using mothur v1.32.1 (Schloss et al., 2009) for subsequent diversity analysis.

All sequence reads were processed for taxonomic assignment by the SILVA NGS analysis pipeline (SILVAngs 1.3, Quast et al., 2013) using SILVA SSU Ref dataset release 119.1 and 123.1.

Raw reads were deposited at the EBI Short Read Archive (SRA) and can be accessed under study accession number SRP070456.

### MasD Amplification, Cloning and Sequencing

The delta subunit of the 1-methyl alkyl succinate synthase gene (*masD*) was amplified from SOFT core DNA (layers 0–1 cm, 2–4 cm, 4–6 cm, 6–8 cm) using the primer pair 7757f-1,f-2 (TCG GAC GCG TGC AAC GMY CTG A; MasD amino acid position 395 in strain HxN1; accession number CAO03074) and 7766f (TGT AAC GGC ATG ACC ATT GCG CT; position 398 in HxN1)/8543r (TCG TCR TTG CCC CAY TTN GG; position 657 in HxN1; von Netzer et al., 2013). For each sample, eight replicate PCRs (20 μl volume) were carried out containing, 1 μM primer each, 250 μM dNTPs, 0.3 μg/μl BSA, 1 × PCR buffer, 0.25 U Taq polymerase (5Prime, Germany) under the following conditions: initial denaturation at 95°C for 5 min, followed by 35 cycles of denaturation (96°C, 1 min), annealing (58°C, 1 min), elongation (72°C, 2 min), and a final elongation step (72°C, 10 min). All replicate PCR reactions per sample were pooled, precipitated with ethanol, and the 800 bp-amplicons were extracted from an agarose gel (1.5% w/v) and purified using the MiniElute PCR Purification Kit (Qiagen) according to the manufacturer’s recommendations. Cloning and Sanger sequencing was performed as described previously in Kleindienst et al. (2012). MasD sequences were clustered at 96% amino acid identity based on 120 amino acid positions (Pos. 398–500, HxN1) using a distance matrix in mother (Schloss et al., 2009).

*MasD* sequences from this study have been deposited in the EMBL, GenBank, and DDBJ nucleotide sequence database under accession numbers LT546441 to LT546456.

### Phylogenetic Analysis

A 16S rRNA-based phylogenetic tree was calculated showing the relationship of described anaerobic hydrocarbon-degrading bacteria to selected reference sequences within the domain Bacteria. The tree was calculated using nearly full-length sequences (>1350 bp) by neighbor joining analysis in combination with filters which consider only 50% conserved regions of the 16S rRNA. Coverage of CARD-FISH probes used in this study are highlighted in the tree.

The MasD phylogenetic tree was calculated by maximum likelihood analysis (PhyML algorithm and Blosum 62 substitution model or Phylip ProML and Dayhoff) with a subset of 280 partial deduced amino acid sequences. Filters were applied considering 196 amino acid positions (Pos. 436–637, strain HxN1) that were conserved in at least 25% of the sequences. The affiliation of MasD sequences retrieved from SOFT core layers is shown together with selected reference sequences from cultured alkane degraders, enrichment cultures, clone libraries or metagenomes. Only one representative sequence per MasD-OTU_0_._96_ is shown in the tree. Unstable branching orders are shown as multifurcations. Sequences from benzylsuccinate synthases (BssA) and naphthyl-methylsuccinate synthases (NmsA) were used as outgroup.

### Diversity Analysis

Using the 16S rRNA gene sequence datasets from untreated and SOFT sediments, the sequence abundance tables after subsampling were used to calculate Inverse Simpson diversity indices and Chao1 richness using mothur v1.32.1 (Schloss et al., 2009). Dissimilarities (Chao, 1984; Hill et al., 2003) were calculated using the Bray–Curtis dissimilarity coefficient (Bray and Curtis, 1957). The resulting beta-diversity matrices were used for three-dimensional non-metric multidimensional scaling (NMDS) ordinations (Kruskal, 1964). Stress values below 0.2 indicate that the multidimensional dataset is well represented by the 3D ordination. To test whether the inclusion of singletons affected further statistical tests we generated NMDS ordinations with and without rare biosphere (OTU comprising <0.01% of total sequences) and compared them using Procrustes correlation analysis (Gower, 1975). Procrustes correlation was highly significant (coefficient = 0.935). Furthermore, to test the effect of subsampling we generated NMDS ordinations using all obtained OTUs and OTUs after subsampling. Procrustes correlation was 0.911. Neither subsampling nor the presence of rare biosphere OTUs did affect the overall trend. Thus we decided to include all data in our analyses, if not stated otherwise, to be able to identify types of microorganisms which can switch from rare to dominant modes of distribution. Taxa that were shared between untreated and SOFT sediments or between layers were calculated using the Jaccard dissimilarity coefficient (i.e., presences/absence). Constrained correspondence analysis (CCA) was carried out to evaluate the combined effects of sulfate, methane, and sulfate reduction rates on the microbial community composition. The significance of these effects was assessed by analysis of similarity (ANOSIM). Environmental factors were fitted in R with the function envfit (vegan package) onto the ordination. The projections of points onto vectors have maximum correlation with corresponding environmental variables, and the factors show the averages of factor levels.

Sequences from untreated-3 years sediments were not included into the diversity analysis because OTU clustering might be biased due to the use of different sequencing platforms.

### Catalyzed Reporter Deposition Fluorescence *In Situ* Hybridization (CARD-FISH)

Sediment samples from the untreated core, untreated-3 years core and SOFT core have been fixed in 3% formaldehyde for 3 h at 4°C, washed with 1x PBS and stored in ethanol-PBS (1:1) at -20°C. Samples were diluted, four times ultrasonicated on ice at 20% intensity, 20 cycles, 30 s (Bandelin Sonopuls HD200), and filtered on a 0.22 μm pore size polycarbonate filter. *In situ* hybridizations with horseradish peroxidase (HRP)-labeled probes followed by fluorescently labeled tyramide signal amplification (catalyzed reporter deposition) were carried out as described previously (Pernthaler et al., 2002). Permeabilization was performed by lysozyme treatment (10 mg ml^-1^) for 60 min at 37°C. Hybridization was done at 46 °C. Hybridized samples were analyzed with an epifluorescence microscope (Nikon Eclipse 50i). For each probe and sample, >1000 DAPI stained cells and their corresponding FISH signals were counted. Used probes (ordered from biomers.net; Ulm, Germany) and formamide concentrations are given in Supplementary Table S1.

## Results and Discussion

### Microbial Diversity in Untreated and SOFT Sediments

We obtained 146,181 bacterial and 393,789 archaeal raw 16S rRNA gene sequences from untreated and SOFT sediments. For bacterial diversity, all depth intervals (0–16 cm depth) were analyzed, while for archaeal diversity the analysis was restricted to the methanogenic zone (6–16 cm depth) to study archaea potentially involved in methanogenic hydrocarbon degradation. Sequencing of bacterial and archaeal 16S rRNA genes were performed by Roche 454 and Ion Torrent technology, respectively. Potential different biases caused by the use of different sequencing platforms are not of relevance here as we handle both datasets separately and do not interpret differences between the bacterial and archaeal diversity. After strict quality trimming about 40% of the raw reads were left for analysis (**Table 1**), with length >430 bp for Bacteria and >310 bp for Archaea. According to the recently revised taxonomic thresholds (Yarza et al., 2014), a total of 2478 bacterial and 153 archaeal genus-level operational taxonomic units (94.5% sequence identity; OTU_0.945_) were detected in the untreated sediment and 2558 bacterial and 241 archaeal OTU_0.945_ in SOFT sediment. The bacterial dataset contained 4% absolute single sequence OTU (SSO_abs_; OTU_0.945_ that occurred only once in the whole dataset) and 11% relative single sequence OTU (SSO_rel_; OTU_0.945_ that occurred only once in at least one sample but are more frequent in other samples) and the archaeal dataset contained 7% SSO_abs_ and 19% SSO_rel_. Chao 1 bacterial genus-level richness estimates based on standardized datasets were similar for untreated and SOFT sediments and ranged between 396 and 532 OTU_0.945_ (**Table 1**). Archaeal richness estimates were one order of magnitude lower and ranged between 30 and 35 OTU_0.945_ in untreated sediments and between 57 and 81 OTU_0.945_ in SOFT sediments. Coverage was >94.6% (Bacteria) and >99.6% (Archaea) for all layers studied and rarefaction curves nearly reaching saturation for most samples indicated sufficient sequencing effort (Supplementary Figure S2). The observed bacterial richness on genus-level is rather low compared to values for species-level diversity reported in other studies investigating benthic habitats, which ranged between ca. 300 OTU_0.97_ at hydrothermal vents to ca. 6500 bacterial OTU_0.97_ at the deep-sea surface and ca. 1500 archaeal OTU_0.97_ in coastal sediments (Ruff et al., 2015). In untreated sediments inverse Simpson for Bacteria ranged between 40.1 and 82.1 and was highest in the two uppermost layers. In SOFT sediments inverse Simpson ranged between 42.9 and 59.9. With an inverse Simpson index between 4.3 and 6.6, archaeal diversity was low in untreated and SOFT sediments (**Table 1**).

**Table 1:** Diversity parameters of Caspian Sea untreated sediments and sediment-oil-flow-through (SOFT) sediments based on next generation sequencing of 16S rRNA genes and Sanger sequencing of cloned *masD*.

Core	Depth [cm]	No. quality reads			No. OTU^a^			No. OTU^b^			Coverage^c^ [%]			Inverse Simpson^b^			Chao 1 richness^b^		
		Bact.	Arch.	MasD	Bact.	Arch.	MasD	Bact.	Arch.	MasD	Bact.	Arch.	MasD	Bact.	Arch.	MasD	Bact.	Arch.	MasD
Untreated	0.5	7256	n.a.	n.d.	552	n.a.	n.a.	438	n.a.	n.a.	97.4	n.a.	n.a.	78.3	n.a.	n.a.	510	n.a.	n.a.
	3	6231	n.a.	n.d.	497	n.a.	n.a.	441	n.a.	n.a.	97.7	n.a.	n.a.	82.1	n.a.	n.a.	443	n.a.	n.a.
	5	3181	n.a.	n.d.	388	n.a.	n.a.	357	n.a.	n.a.	94.7	n.a.	n.a.	60.4	n.a.	n.a.	400	n.a.	n.a.
	7	2798	6536	n.d.	346	29	n.a.	315	29	n.a.	94.6	99.86	n.a.	44.0	4.6	n.a.	396	30	n.a.
	9	n.a.	3931	n.a.	n.a.	30	n.a.	n.a.	30	n.a.	n.a.	99.77	n.a.	n.a.	5.7	n.a.	n.a.	33	n.a.
	11	3053	6256	n.a.	365	31	n.a.	340	29	n.a.	94.9	99.84	n.a.	57.5	4.6	n.a.	497	35	n.a.
	13	n.a.	3908	n.a.	n.a.	32	n.a.	n.a.	32	n.a.	n.a.	99.69	n.a.	n.a.	6.0	n.a.	n.a.	34	n.a.
	15	2730	5337	n.a.	330	31	n.a.	330	31	n.a.	95.0	99.83	n.a.	40.1	6.0	n.a.	470	34	n.a.
SOFT	0.5	4229	n.a.	176	456	n.a.	2	411	n.a.	2	96.2	n.a.	99.4	47.9	n.a.	1.0	532	n.a.	22
	3	6412	n.a.	192	489	n.a.	5	398	n.a.	3	97.2	n.a.	99.0	59.9	n.a.	1.2	508	n.a.	5
	5	6588	n.a.	187	416	n.a.	10	401	n.a.	6	98.1	n.a.	96.8	44.6	n.a.	2.3	496	n.a.	6
	7	5280	28709	162	391	48	7	364	32	3	97.6	99.97	97.5	49.9	4.3	1.3	548	69	7
	9	n.a.	22394	n.a.	n.a.	46	n.a.	n.a.	38	n.a.	n.a.	99.97	n.a.	n.a.	5.4	n.a.	n.a.	81	n.a.
	11	3247	15661	n.a.	354	43	n.a.	332	30	n.a.	95.5	99.95	n.a.	42.9	6.1	n.a.	520	57	n.a.
	13	n.a.	29369	n.a.	n.a.	52	n.a.	n.a.	32	n.a.	n.a.	99.95	n.a.	n.a.	4.8	n.a.	n.a.	77	n.a.
	15	6185	31960	n.a.	452	52	n.a.	426	36	n.a.	97.5	99.97	n.a.	58.6	6.6	n.a.	452	62	n.a.

*^a^Operational taxonomic units clustering for bacterial and archaeal 16S rRNA gene sequences was done on genus-level using a threshold of 94.5% sequence identity (OTU_*0.945*_); clustering for MasD was done on species-level using a threshold of 96% amino acid identity (MasD OTU_*0.96*_). ^b^Standardized based on resampling without replacement of 2730 sequences for bacterial 16S rRNA, 3908 sequences for archaeal 16S rRNA and 162. ^c^Library coverage (C) calculated per subsample as C = [1 - (n/N)]^∗^100 (Good, 1953), where n is the number of singletons (SSOabs + SSOrel), and N is the number of sequences analyzed. n.a., not analyzed; n.d., no PCR product obtained.*

### Microbial Community Structure in Untreated and SOFT Sediments

The uppermost layer of the SOFT core (0–1 cm depth) was excluded from similarity analysis due to the influence of an accumulated oil slick above and oxygen penetration into this layer.

Pairwise comparison of untreated and SOFT sediments resulted in 43% shared bacterial OTU_0.945_ but only 23% shared archaeal OTU_0.945_ (Supplementary Table S2). Communities were as similar to each other within the core as between identical layers of the two cores: On average, 55% bacterial and 59% archaeal OTU_0.945_ were shared within a core versus 51–56% bacterial and 59% archaeal OTU_0.945_ shared between identical layers (Supplementary Table S3).

**FIGURE 2:**
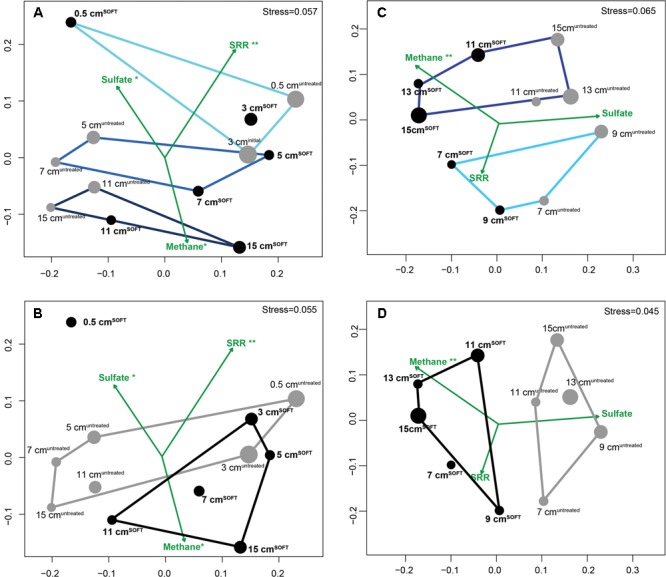
**Similarity of bacterial (A,B)** and archaeal **(C,D)** communities visualized by non-metric multidimensional scaling (NMDS) of Bray–Curtis dissimilarity matrices based on OTU_0.945_ relative abundance data with environmental variables fitted as vectors. Gray dots represent untreated sediment subsamples and black dots represent SOFT sediment subsamples. The size of the dots reflects the evenness of the subsamples (inverse Simpson index). Defined subgroups are depicted as polygons. Dataset for SOFT core layer 0.5 cm depth was excluded from the analysis because this layer became oxic and was influenced by an oil slick forming on top of the core during the experiment. Significance levels are indicated next to the tested parameters methane, sulfate, and sulfate reduction rates (^∗∗^*p* < 0.01; ^∗^*p* < 0.1). SRR, sulfate reduction rates.

Similarity of bacterial (**Figures 2A,B**; 11 samples) and archaeal (**Figures 2C,D**; 10 samples) communities was visualized by NMDS. Bacterial dissimilarity (Bray–Curtis) of samples is supported by an *R* value of 0.49 (*p* < 0.001). Archaeal dissimilarity (Bray–Curtis) of samples is supported by an *R* value of 0.57 (*p* < 0.001). The bacterial and archaeal community differed significantly between different sediment layers as confirmed by ANOSIM (**Figures 2A,C**; Bacteria: *R* = 0.46–0.73, *p* < 0.001; Archaea: *R* = 0.75; *p* < 0.001). Between the cores, community was only significantly different for Archaea (*R* = 0.73; *p* < 0.001) but not for Bacteria (*R* = 0.31, *p* = 0.05; **Figures 2B,D**).

Fitting of environmental variables showed significant correlations for methane (*p* = 0.003), suggesting that methane likely influenced the observed pattern of archaeal taxonomic clustering, while sulfate reduction rates (*p* < 0.001) and sulfate concentration (*p* = 0.07) likely influenced bacterial taxonomic clustering. As mentioned earlier, replication of the SOFT experiment was not feasible; however, we consider adjacent layers of each core as technical replication, providing a certain confidence level for the comparison between the untreated and the SOFT sediments.

### Bacterial Taxa in Untreated and SOFT Sediments

**FIGURE 3:**
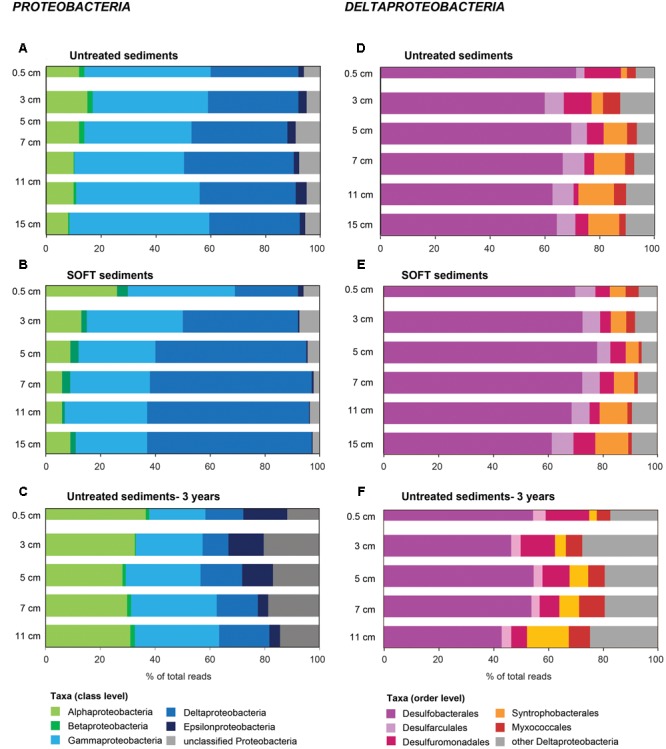
**Community composition of (A–C)** *Proteobacteria* and **(D–F)** *Deltaproteobacteria* in untreated **(A,D)**, untreated-3 years **(C,F)** and SOFT **(B,E)** sediments from Caspian Sea based on 16S rRNA gene sequencing. Petroleum flow-through resulted in a community shift in SOFT sediments toward a higher percentage of deltaproteobacterial sequences. Storage of untreated sediments for 3 years at 0°C in the dark **(C,F)** resulted in a shift toward *Alphaproteobacteria* and *Epsilonproteobacteria* and a decrease of *Deltaproteobacteria* indicating that long-term storage did not favor the growth of deltaproteobacterial, hydrocarbon-degrading sulfate reducers identified in this study.

On phylum-level, the composition of the bacterial community was similar in untreated and SOFT sediments (Supplementary Figures S3A,B). Proteobacteria dominated throughout the cores accounting for 56 and 50% of bacterial 16S rRNA gene sequences in untreated and SOFT sediments, respectively. Planctomycetes, Actinobacteria, and Chloroflexi (5–7% each) were the next sequence-abundant phyla. Within Proteobacteria, one-third (untreated sediment) and one-half (SOFT) of the sequences were assigned to the class Deltaproteobacteria (**Figures 3A,B**) while almost one-half (untreated sediment) and one-third (SOFT) were assigned to Gammaproteobacteria. In comparison, previous studies investigating the microbial community in natural Caspian Sea sediments reported similar taxa with Proteobacteria being most abundant (33% of reads on average), followed by Planctomycetes (14%) and Chloroflexi (12%, Mahmoudi et al., 2015).

The major part of gammaproteobacterial sequences in SOFT and untreated sediment affiliated with Woeseiaceae/JTB255 (29 and 30%), recently described as ubiquitous chemolithoautotrophic key players potentially involved in sulfur oxidation in marine sediments (Dyksma et al., 2016). Alphaproteobacterial genera described to aerobically degrade aromatic hydrocarbons (Kim and Kwon, 2010), e.g., *Novosphingobium, Sphingomonas, Thalassospira*, and *Kordiimonas*, have been found in the SOFT dataset retrieved from 0.5 cm depth. As the top layer in the SOFT core was oxic and was covered by an oil slick, the detection of sequences from known hydrocarbon degraders suggested the development of an aerobic hydrocarbon-degrading bacterial community. Furthermore, sequences related to known aerobic gammaproteobacterial hydrocarbon degraders (Kleindienst et al., 2016) have also been detected in this layer including *Cycloclasticus, Marinobacter, Alcanivorax*, and *Thalassolituus*. In particular, the relative abundance of sequences assigned to *Cycloclasticus* increased remarkably by a factor of 10 from 0.1% in untreated sediments to 1.0% in the oxic SOFT layer. *Cycloclasticus* spp. have been repeatedly isolated from marine sediments and were shown to use aromatic hydrocarbons such as naphthalene, phenanthrene, anthracene, fluorenes, and toluene as sole carbon source (Dyksterhouse et al., 1995; Geiselbrecht et al., 1998; Chung and King, 2001; Cui et al., 2014). Likewise, culture-independent studies supported that *Cycloclasticus* spp. likely play a major role in the degradation of petroleum polycyclic aromatic hydrocarbons in coastal (Coulon et al., 2012), deep-sea (Cui et al., 2008; Dong et al., 2015; Gutierrez et al., 2015), and estuarine (McKew et al., 2007; Niepceron et al., 2010) sediments.

Methanogenesis and sulfate reduction were identified as important processes in the anaerobic degradation of hydrocarbons during petroleum seepage in Caspian Sea sediments (Mishra et al., this issue). Therefore, Deltaproteobacteria were analyzed in greater detail because most known anaerobic hydrocarbon degraders belong to this class (**Figure 1**). A slight increase of relative sequence abundance of Deltaproteobacteria was observed in the anoxic layers of the SOFT core between 2 and 8 cm depth (17–25% in untreated sediments versus 23–35% in SOFT sediment **Figures 3D,E**). Of these, more than one-half could be further assigned to the order Desulfobacterales with highest frequency between 4 and 12 cm depth (**Figure 3E** and Supplementary Table S4). Within Desulfobacterales, the uncultured Sva0081-group, which was first described in clone libraries from Svalbard sediments (Ravenschlag et al., 1999) and includes sulfate-reducing endosymbionts of the gutless marine oligochaete *Olavius* sp., was the most sequence-abundant group with 2.3–5.7% of total sequences (Supplementary Table S4). Relative abundance did not differ remarkably between untreated and SOFT sediments supporting current knowledge, that Sva0081 bacteria are ubiquitous and highly abundant members of SRB communities in diverse benthic habitats (Mußmann et al., 2015). A second sequence-abundant group was the uncultivated clade SEEP1d with up to 3.4% of total bacterial sequences between 10 and 16 cm in both, untreated and SOFT sediments. SEEP1d was repeatedly found at marine seep sites but no function has yet been assigned (Knittel et al., 2003; Schreiber et al., 2010).

Many known groups of hydrocarbon-degrading SRB (**Figure 1**) were found in our dataset, some of which showed an increase of relative sequence abundance after oil-flow-through in the zone of highest sulfate reduction at 2–8 cm SOFT depth; other did not (Supplementary Table S4). Most sequence-abundant and at the same time strongly increased were relatives of *Desulfobacula* with up to 8.1% of total bacterial sequences compared to 1.7% in untreated sediments. *Desulfobacula*-related sequences retrieved from SOFT sediments showed 96% sequence similarity with *D. toluolica* and *D. phenolica*. *Desulfobacula* spp. can use short-chain fatty acids and simple organic compounds as electron donors but also various aromatic compounds such as toluene or phenol (Bak and Widdel, 1986; Rabus et al., 1993; Kuever et al., 2001). Putative long-chain alkane-degrading SRB of clade LCA2 (Kleindienst et al., 2014, Figure 1) were less sequence-abundant but increased in SOFT sediments, with 0.2% vs. 1.9%, as well as clade Cyhx (Jaekel et al., 2015, 0.2% vs. 0.6%), suggested C_2_–C_4_ alkane degraders (Adams et al., 2013, 0.1% vs. 1.1%), *Desulfosarcina* spp. (0.6% vs. 1.5%), and s2551group (0.5% vs. 1.1%), reported as oil-degrading bacteria in an oil-reservoir model column (Myhr et al., 2002). Relative sequences abundances of putative short-chain alkane-degrading clade SCA1, which comprises the only isolated *n-*butane-degrading strain BuS5 (Kniemeyer et al., 2007), did not increase after oil-flow-through and were constantly low with 0.3–0.8% of total bacterial sequences in both, untreated and SOFT sediments. No remarkable change (0.2% vs. 0.3%) has also been observed for sequences affiliated with *Desulfococcus oleovorans* Hxd3 (Aeckersberg et al., 1991), clade LCA1 (0.1%), *Desulfatiglans*-group (1.1–2.1%), and clade SB-29 (around 0.5%). Sequences related to known hydrocarbon-degraders within the Firmicutes, i.e., Propane60GuB (Kniemeyer et al., 2007) and *Desulfotomaculum* sp. strain Ox39 (Morasch et al., 2004), were not retrieved. However, relative abundance of sequences within Peptococcaceae, in particular those related to autotrophic hydrogen-utilizing SRB of the genus *Desulfosporosinus* spp., increased at 15 cm depth in SOFT sediments (untreated sediments: 1.1%; SOFT: 4.8% of total bacterial sequences).

Due to logistical complications, the SOFT core was stored for three more months after sampling and processing the untreated sediments. To verify that observed changes in the SRB community in SOFT sediments were independent of storage effects but caused by oil-flow-through, a parallel core stored for 3 years at 0°C in the dark (“untreated-3 years”) was analyzed by Illumina tag sequencing. After strict quality trimming, 42982 bacterial 16S rRNA gene sequences were taxonomically assigned using the SILVAngs pipeline. The composition of the microbial communities was compared on class level to avoid potential bias due to the use of different sequencing platforms. Furthermore, we restricted the comparison to Proteobacteria because most sulfate reducers detected in SOFT sediments belonged to this phylum. Storage of untreated sediments for 3 years at 0°C in the dark resulted in a shift toward Alphaproteobacteria (8–15% of total bacterial sequences in untreated vs. 26–36% in untreated-3 years sediments; mainly Rhodobacterales and Rhodospirillales) and Epsilonproteobacteria (2–4% vs. 4–16%; mainly *Sulfurovum* and *Sulfurimonas*) and a decrease of Deltaproteobacteria (32–40% vs. 9–18%; **Figures 3C,F**). We therefore conclude that the observed increase of relative sequence abundance of hydrocarbon-degrading deltaproteobacterial SRB in SOFT sediments points to a positive effect of petroleum on the growth of this bacterial group in the oil-flow-through system rather than being caused by storage effects.

### Archaeal Taxa in Untreated and SOFT Sediments

On phylum-level, significant differences were observed between the archaeal community in SOFT and untreated sediments (Supplementary Figures S3C,D). In untreated sediments at 7 and 9 cm depth, most sequence-abundant phylum was Thaumarchaeota (on average 30% of total archaeal sequences at 7–15 cm depth with a maximum of 64% at 7 cm), of which all currently known species are chemolithoautotrophic ammonium-oxidizers (Könneke et al., 2014). Below, at 11–15 cm depth, sequences related to euryarchaeotal Marine Benthic Group D and Deep-sea Hydrothermal Vent Euryarchaeotic Group (DHVEG-1; 20% of total archaeal sequences) and Thermoplasmata CCA47 (13%), a group of uncultivated euryarchaeotal sequences isolated from an anoxic sediment of a sub-saline shallow lake (Laguna de Carrizo, Central Spain, Ferrer et al., 2011), were most abundant. In the SOFT core, Thaumarchaeota had only low sequence frequencies (0.6–2.5%). Dominant groups were DHVEG, Thermoplasmata ASC21 (9%) a group of uncultivated Thermoplasmatales isolated from a hot spring in the Lower Culex region of the Lower Geyser Basin, Yellowstone National Park (Saw et al., 2015), and Aenigmarchaeota of the Deep Sea Euryarchaeotic Group (DSEG; 7% in 14–16 cm). High relative sequence numbers of Methanosarcinales were detected at 7 cm (7% of total archaeal sequences) and at 9 cm (38%) depth in the SOFT core, whereas in other layers and in untreated sediments they were nearly absent. More than 99% of the Methanosarcinales sequences affiliated with the genus *Methanosarcina*. The closest relatives were *M. semesiae* and *M. lacustris*, with a 16S rRNA gene similarity of >98%. Furthermore, only very few sequences (<<1% of total archaeal sequences) affiliated with other known methanogens such as *Methanolobus* and *Methanococcoides* (Methanosarcinaceae), Methanosaetaceae, Methanomicrobiaceae, Methanocellaceae, or Methermicoccaceae were detected in SOFT sediments (Supplementary Table S5). Methanotrophs of the ANME clades (ANME-1, ANME-2, ANME-3) and “*Candidatus* Syntrophoarchaeum,” capable of anaerobic butane oxidation (Laso-Pérez et al., 2016) were only sporadically detected. In comparison, previous studies of the microbial community in Caspian Sea sediments identified Thaumarchaeota and Parvarchaeota as dominant in surface layers while Marine Benthic Group B (Lokiarchaeota) dominated the deeper layers (Mahmoudi et al., 2015).

### Response of Hydrocarbon-Degrading SRB to Simulated Petroleum Seepage

**FIGURE 4:**
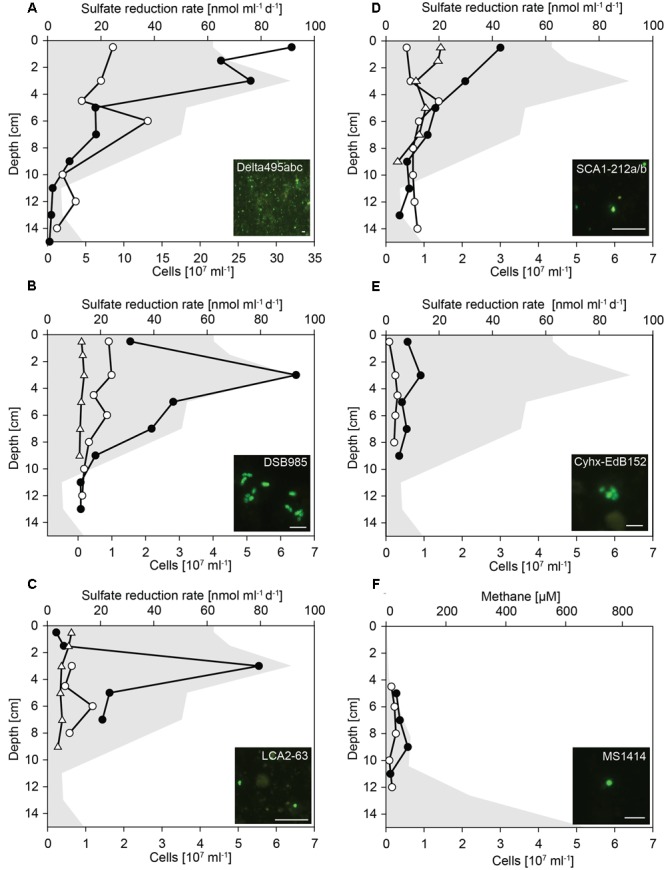
**Cell numbers of Deltaproteobacteria and hydrocarbon-degrading SRB in untreated and SOFT sediments.** Vertical profiles show specific cell numbers in untreated (white dots) and SOFT (black circles) core sediments as detected by CARD-FISH with **(A)** probe Delta495abc for Deltaproteobacteria, **(B)** probe DSB985 for *Desulfobacula* spp., *Desulfotignum* spp., and *Desulfobacter* spp., **(C)** probe LCA2-63 for clade LCA2, **(D)** probe SCA1-212a/b for clade SCA1, **(E)** probes Cyhx-EdB_152/152b for clade Cyhx, and **(F)** probe MS1414 for *Methanosarcina* spp., *Methanococcoides* spp., *Methanolobus* spp., and *Methanohalophilus* spp. As reference, cell numbers detected with probes SCA1-212a/b, DSB985, and LCA2-63 were determined in the untreated-3 years core that was stored for 3 years at 0°C in the dark (white triangles). Sulfate reduction rates **(A–E)** and methane concentration **(F)** in SOFT core sediments are visualized by gray area (data taken from Mishra et al., this issue). Bar = 5 μm.

Based on identified changes in relative sequence abundance, we selected several groups of SRB as target for quantification by CARD-FISH (**Figure 4**).

Deltaproteobacteria were most abundant between 0 and 4 cm depth (**Figure 4A**). In these layers, deltaproteobacterial cell numbers increased by a factor of 4 compared with numbers in untreated sediment layers and accounted for up to 3.2 × 10^8^ cells ml^-1^ SOFT sediment (24–28% of total cells) and up to 8.6 × 10^7^ cells ml^-1^ untreated sediment (9–17%). The peak of Deltaproteobacteria in SOFT sediment coincided with the strong decrease of sulfate concentration, highest sulfate reduction rates and the decrease of aliphatic hydrocarbon concentrations (see Mishra et al., this issue) indicating a response of specific hydrocarbon-degrading Deltaproteobacteria. Major decrease of gaseous alkanes was observed above 7 cm depth (Mishra et al., this issue). Mid- and long-chain alkanes (C_12_–C_30_) decreased by about 50% above 14 cm depth, further 25% above 7 cm and remained relatively constant until top of the core.

Using probe DSB985, we targeted *Desulfobacula, Desulfotignum*, and *Desulfobacterium*, genera comprising aromatic hydrocarbon degraders. Members of this target group were detected in high numbers in the SOFT core accounting for up to 6.4 × 10^7^ cells ml^-1^ sediment (7% of total cells, **Figure 4B**). Cell numbers peaked at 3 cm depth and strongly decreased below. In untreated sediments, *Desulfobacula* cell numbers were almost an order of magnitude lower accounting for only 1% of total cells. Similar low cell numbers were observed in untreated sediments stored for 3 years. Although degradation of aromatic compounds had not been followed during the experiment, we assume a standard composition of the light oil used for flow-through of about 30% aromatics, 0–10% asphaltenes, and major part being alkanes. Highest cell numbers of *Desulfobacula*-related species were found within the zone of sulfate reduction thus suggesting that this group might be the main consumer of the aromatics in the petroleum.

The yet uncultivated LCA2-group was targeted by probe LCA2-63. Like *Desulfobacula*, LCA2 cell numbers were highest at 3 cm depth in SOFT sediments with 5.5 × 10^7^ cells ml^-1^ sediment (6% of total cells). In both untreated sediments, LCA2 cell numbers were one order of magnitude lower (**Figure 4C**) and accounted for only 1% of total cells. SRB of LCA2 had been identified by stable isotope-probing as key players for long-chain alkane degradation at marine seeps (Kleindienst et al., 2014). The incubation was done with *n-*dodecane as a representative substrate for long-chain alkanes. Here, SRB of LCA2 are suggested as putative main consumers of long-chain alkanes in the 2–6 cm depth layers in SOFT sediment. Accordingly, concentrations of C_12_–C_26_ alkanes dropped to about 20–25% in these depth layers (Mishra et al., this issue).

Short-chain alkane-degrading clade SCA1 was targeted by probes SCA1-212a and SCA1-212b. SCA1 cell numbers were comparably low for untreated, untreated-3 years and SOFT sediment layers. Only in SOFT sediment at 0–3 cm depth, SCA1 cell numbers were 3- to 5-fold higher than in both control sediments (2–3 × 10^7^ cells ml^-1^; 2% of total cells; **Figure 4D**). Isolated or enriched members of SCA, for example strain BuS5, Butane12_GMe, propane12_GMe, and Butane12_HR, use propane or butane as carbon source (Kniemeyer et al., 2007; Jaekel, 2011; Jaekel et al., 2013). We suggest that SCA1 together with others (for example, the C_2_–C_4_ alkane-degrading group, Adams et al., 2013) are the main consumers of available propane and butane in the SOFT sediment.

Clade Cyhx was targeted by a mix of probe Cyhx28_EdB_152 (Jaekel et al., 2015) and probe Cyhx28_EdB_152b that has been designed in this study. In the zone of sulfate reduction in the SOFT core, representatives of this clade were detected by CARD-FISH in numbers of up to 0.9 × 10^7^ cells ml^-1^ sediment (1% of total cells, **Figure 4E**). In all layers of untreated sediments, members of clade Cyhx were close to detection limit of ≤0.5% of total cells. Cyhx-SRB were shown to grow on cyclohexane, but also to use other cyclic alkanes as well as *n*-pentane and *n*-hexane (Jaekel et al., 2015).

### Methanogenic Hydrocarbon Degradation as Response to Simulated Oil Seepage

Methanogenesis was identified as important process in the anaerobic degradation of hydrocarbons during simulated oil seepage in SOFT sediments. The δ^13^C signal of produced methane showed a decrease from -33.7 to -49.5 after 190 days of oil seepage indicating microbial methane formation (Mishra et al., this issue). Furthermore, the high methane concentrations in the deep SOFT core layers coincided with a decrease in higher hydrocarbons also supporting methanogenic hydrocarbon degradation. The mechanism driving alkane (mainly hexane) or aromatic hydrocarbon degradation under methanogenic condition is not elucidated yet. It supposedly requires the interaction of syntrophic bacteria, such as members of the family Syntrophaceae (*Syntrophus* spp., *Smithella* spp.) or of the order Clostridiales (*Desulfotomaculum* spp.), with methanogenic archaea (Zengler et al., 1999; Siddique et al., 2012). For example, *Methanosaeta* spp. and *Methanoculleus* spp. have been repeatedly detected in methanogenic hydrocarbon-degrading enrichment cultures (Siddique et al., 2006, 2011; Cheng et al., 2013). For *in situ* identification and quantification of methanogens in the SOFT core methanogenic zone we applied probe MS1414 targeting *Methanosarcina, Methanococcoides, Methanohalophilus, and Methanolobus*. Cell numbers were highest with 5.7 × 10^6^ cells ml^-1^ (2% of total cells) at the depth of 9 cm (**Figure 4F**) where maximum methane increase was observed. Although hydrocarbon-degradation by *Methanosarcina* spp., which are the dominant methanogens in this study, has not been reported yet, and although detected *Methanosarcina* cells were not physically attached to any other cell, we hypothesize a contribution of this group to observed methanogenic hydrocarbon-degradation due to their specific increase in cell numbers in the methanogenic zone. The well-known bacterial partners such as *Syntrophus* or *Desulfotomaculum* were absent from the dataset. Only sporadically, *Smithella* sequences (*n* = 2 at 13 cm depth) were found. Syntrophic growth of *Methanosarcina* has been described for an association with *Geobacter* spp. and depends on interspecies electron transfer (Rotaru et al., 2014), thus also allowing the speculation about hydrocarbon-degradation with a yet unknown bacterial partner.

### Anaerobic Alkane-Degrading Community Based on masD Gene Diversity

The microbial community that activates alkanes by fumarate addition was studied by cloning of *masD* genes from selected SOFT core sediment layers that showed the highest sulfate reduction activity. Bss that activate aromatic compounds were not matched by the used primer pair. Considering the threshold for MasD species level of 96% amino acid identity recently defined by Stagars et al. (2016), a total of 16 species-level OTU_0.96_ were retrieved after sequencing of 717 *masD*-carrying clones. From untreated sediments, *masD* could not be amplified. Library coverage was >96.8% for all samples. With inverse Simpson values of close to 1 (1.0–2.3), diversity of MasD was very low in SOFT core sediments (**Table 1**). As comparison, the MasD diversity was even lower than in 12 globally distributed hydrocarbon seep sites for which inverse Simpson values of 3–9 were reported (Stagars et al., 2016).

**FIGURE 5:**
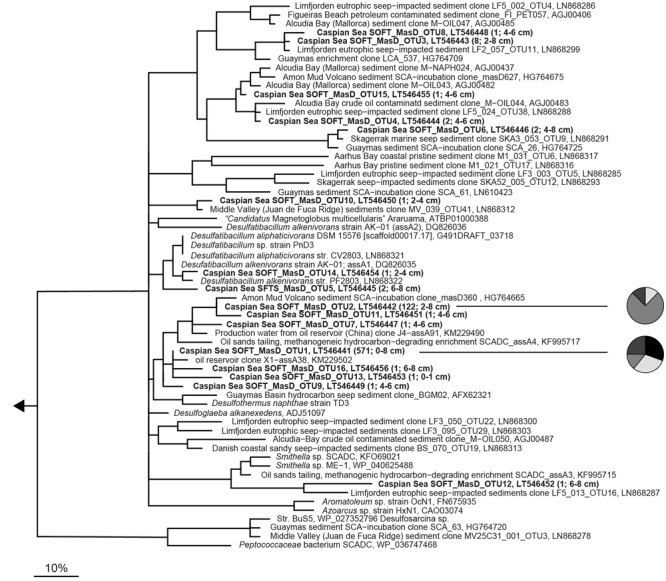
**Phylogeny of partial MasD/AssA sequences retrieved from SOFT core sediments and selected reference sequences.** Operational taxonomic units (OTU) were defined at a cut-off of 96% amino acid identity (according to Stagars et al., 2016). Representative sequences for OTUs from SOFT sediments are printed in bold. OTU abundance (number of sequences) and sediment depth are indicated in parenthesis. The scale bar gives 10% estimated amino acid sequence divergence. Pie charts shown next to the two most abundant OTUs give the distribution in different depth layers. Black, 0–1 cm; white, 2–4 cm; medium gray, 4–6 cm, and dark gray, 6–8 cm depth.

MasD OTU_0.96_ were widely distributed over the phylogenetic tree suggesting the presence of diverse anaerobic alkane-degrading bacteria in the sulfate reduction zone of SOFT sediments (**Figure 5**). However, two MasD OTU_0.96_, i.e., MasD_OTU1 and OTU2, contained 97% (571 sequences and 122 sequences) of all retrieved MasD sequences. They were both assigned to a bigger cluster that comprised *Desulfothermus naphthae* str. Td-3, a mid-chain *n*-alkane (C_6_–C_16_)-degrading sulfate-reducing bacterium isolated from oily Guaymas Basin sediments (Rueter et al., 1994) as the only cultivated representative. OTU1 and OTU2 were most closely affiliated to a sequence from a butane enrichment from Amon mud volcano seep sediments (Kleindienst et al., 2014) and to a clone sequence from production water from a Chinese oil reservoir (Bian et al., 2014; database release). Our CARD-FISH data pointed to a response of SCA1/BuS5 on the simulated petroleum seepage. BuS5-related MasD sequences, however, were not found. This is most likely caused by substantial primer mispairing: The forward primers had 11 (7757f1-f2, 22mer) and 13 (7766f, 23mer) mismatches, respectively, to the BuS5-*masD* sequence retrieved from the genome (JGI gene ID 2513990058). Our MasD data support the recent findings by Stagars et al. (2016) who detected a total of 420 MasD-carrying species in 12 different hydrocarbon seep environments of which only few were abundant, cosmopolitan alkane degraders but many were specialized taxa across many different environments. It also matches our findings that short-chain alkane-degrading group SCA1 and long-chain alkane-degrading group LCA2 were the only taxa identified, which responded to simulated petroleum seepage by an increase of their cell numbers suggesting that we likely have identified the key alkane-degrading SRB in Caspian Sea sediments.

## Conclusion

By molecular comparison of microbial communities in Caspian Sea sediments before and after controlled petroleum seepage, specific SRB and methanogenic archaea were identified who are likely responsible for the observed decrease in aliphatic hydrocarbon concentration. Although no aliphatic hydrocarbons could be detected in untreated Caspian Sea sediment, this study demonstrates the intrinsic potential for alkane degradation in the microbial community most likely due to a history of nearby hydrocarbon seepage. This study further suggests that identified SRB, such as members of the clades SCA1, LCA2, *Desulfobacula*, and Cyhx, are likely responsible for hydrocarbon degradation under close-to-in situ conditions. For some groups, cultivation has already been successful (BuS5, Kniemeyer et al., 2007); *Desulfobacula* (Rabus et al., 1993; Heider et al., 1998), and others have been enriched (clade Cyhx). Similar to aerobic hydrocarbon degradation in the water column, for example after the *Deepwater Horizon* accident, where gammaproteobacterial *Oceaniserpentilla, Colwellia*, or *Cycloclasticus* dominated in the hydrocarbon plume (Kleindienst et al., 2016), the major part of anaerobic hydrocarbon degradation is mediated by few groups of microbes specialized on the degradation of a specific hydrocarbon type: SCA1 is suggested to be likely responsible for propane and butane degradation [maybe together with the C_2_–C_4_ short-chain alkane-degrading group described by Adams et al. (2013)], LCA2 is suggested to be involved in mid- to long-chain alkane degradation, membes of clade Cyhx are suggested to be responsible for cycloalkanes, pentane, and hexane degradation, relatives of *Desulfobacula* are suggested as consumers of aromatic compounds and archaea of the genus *Methanosarcina* might be responsible for syntrophic methanogenic long-chain alkane degradation. Isolation attempts, metatranscriptomic and metagenomic approaches as well as single cell analyses of probe-targeted cells will help to get a deeper understanding of the ecophysiology of these clades and their suggested role in anaerobic hydrocarbon degradation.

## Author Contributions

KK, TT, and RA designed the study. SM and TT planned and conducted the SOFT experiment. SM did the field sampling and sulfate reduction measurements. MS processed tag sequencing data, analyzed the microbial diversity, constructed masD gene libraries and performed phylogenetic analysis of MasD. KK performed CARD-FISH experiments and cell counting as well as phylogenetic analysis of 16S rRNA genes from hydrocarbon degraders. MS, TT, RA, and KK wrote the manuscript with input from SM.

## Conflict of Interest Statement

The authors declare that the research was conducted in the absence of any commercial or financial relationships that could be construed as a potential conflict of interest.
