# Supplementary material for: Microbial Community Response to Simulated Petroleum Seepage in Caspian Sea Sediments
Source: Front Microbiol. 2017 Apr 28;8:764. doi: 10.3389/fmicb.2017.00764 (PMC5409227; doi:10.3389/fmicb.2017.00764)
Supplement: Supplementary file 1 [file Presentation_1.PDF]

Supplementary Information

Microbial community response to simulated petroleum seepage in Caspian Sea sediments

Marion Stagars[1], Sonakshi Mishra[2], Tina Treude[2,3], Rudolf Amann[1], and Katrin Knittel[1*]

Affiliations

1 Max Planck Institute for Marine Microbiology, Bremen, Germany

2 GEOMAR Helmholtz Center for Ocean Research Kiel, Kiel, Germany

3 Department of Earth, Planetary and Space Sciences, Department of Atmospheric and Oceanic Sciences, University of California, Los Angeles, CA, USA

Stagars et al.

Table S1. Oligonucleotide probes used in this study

Probe name	Specificity	Form-amide [%]	Sequence (5' - 3')	Reference
SCA1-212a	SCA-SRB1	20	CATCCCAAAACAGTAGCT	Kleindienst *et al*., 2014
SCA1-212b	SCA-SRB1	20	CATCCCCAAACAGTAGCT	
h1_SCA1-197	Helper for SCA1-212ab		TATWTATAGAGGCCA	
h2_SCA1-197			TATAWATAGAGGCCA	
h3_SCA1-182			CCTTTGATCTRAAAA	
h4_SCA1-182			CCTTTGATCTGAAWA	
h5_SCA1-229			GCTAATGGTACGCGRGCT	
h6_SCA1-182			CCTTTGATCTGGATA	
LCA2-63	LCA2	10	GCUAAAGCUUUCUCGUUC	Kleindienst *et al*., 2014
h1_LCA2-83	Helper for LCA2-83		CUUUACUCACUCUAGCAA	
Cyhx28-EdB_152	Clade Cyhx	20	ACGAAGCCTTTCAGCATG	Jaekel *et al*., 2015
Cyhx28-EdB_152_mod	Clade Cyhx	20	ACGAAGCCTTTCGGCATG	This study
DSB985	*Desulfobacter*, *Desulfobacula*, *Desulfospira*, *Desulfotignum*	20	CACAGGATGTCAAACCCAG	Manz *et al*., 1998
Arch915	Archaea	35	GTGCTCCCCCGCCAATTCCT	Stahl *et al*., 1988
Delta495a	*Deltaproteobacteria*	30	AGTTAGCCGGTGCTTCCT	Loy *et al*.,2002
Delta495b			AGTTAGCCGGCGCTTCCT	
Delta495c			AATTAGCCGGTGCTTCCT	
cDelta495a	Helper for Delta495	30	AGTTAGCCGGTGCTTCTT	
cDelta495b			AGTTAGCCGGCGCTTCKT	
cDelta495c			AATTAGCCGGTGCTTCTT	
Non338	Negative control	35	ACTCCTACGGGAGGCAGC	Wallner *et al*., 1993
MS1414	*Methanosarcinales*	50	CTCACCCATACCTCACTCGGG	Crocetti *et al*., 2006
hMS1395	Helper for MS1414		GGTTTGACGGGCGGTGTG	
hMS1480			CGACTTAACCCCCCTTGC	

References

Crocetti, G., Murto, M., and Björnsson, L. (2006) An update and optimisation of oligonucleotide probes targeting methanogenic Archaea for use in fluorescence in situ hybridisation (FISH). *Journal of Microbiological Methods* **65**: 194-201.

Jaekel, U., Zedelius, J., Wilkes, H., and Musat, F. (2015) Anaerobic degradation of cyclohexane by sulfate-reducing bacteria from hydrocarbon-contaminated marine sediments. *Frontiers in Microbiology* **6**: 116.

Kleindienst, S., Herbst, F.A., Stagars, M., von Netzer, F., von Bergen, M., Seifert, J. et al. (2014) Diverse sulfate-reducing bacteria of the Desulfosarcina/Desulfococcus clade are the key alkane degraders at marine seeps. *ISME Journal* **8**: 2029-2044.

Loy, A., Lehner, A., Lee, N., Adamczyk, J., Meier, H., Ernst, J. et al. (2002) Oligonucleotide Microarray for 16S rRNA Gene-Based Detection of All Recognized Lineages of Sulfate-Reducing Prokaryotes in the Environment. *Applied and Environmental Microbiology* **68**: 5064-5081.

Manz, W., Eisenbrecher, M., Neu, T.R., and Szewzyk, U. (1998) Abundance and spatial organization of gram-negative sulfate-reducing bacteria in activated sludge investigated by in situ probing with specific 16S rRNA targeted oligonucleotides. *Fems Microbiology Ecology* **25**: 43-61.

Stahl, D.A., Flesher, B., Mansfield, H.R., and Montgomery, L. (1988) Use of phylogenetically based hybridization probes for studies of ruminal microbial ecology. *Applied and Environmental Microbiology* **54**: 1079-1084.

Wallner, G., Amann, R., and Beisker, W. (1993) Optimizing fluorescent in situ hybridization with rRNA-targeted oligonucleotide probes for flow cytometric identification of microorganisms. *Cytometry* **14**: 136-143.

Table S2: Pairwise comparison of community similarity within groups of samples based on presence-absence of A) bacterial and B) archaeal 16S rRNA $OTU_{0.945}$ of standardized data. Percentage of shared bacterial and archaeal $OTU_{0.945}$ between groups of samples is given.

**A**		SOFT 0-16 cm	untreated 4-8 cm	untreated 10-16 cm	SOFT 0-4 cm	SOFT 4-8 cm	SOFT 10-16 cm
**Bacteria**	untreated 0-16 cm	43					
	untreated 0-4 cm		36	31	56	38	42
	untreated 4-8 cm			33	39	51	37
	untreated 10-16 cm				33	36	61
	SOFT 0-4 cm					41	31
	SOFT 4-8 cm						35

**B**		SOFT 6-16 cm	untreated 10-16 cm	SOFT 6-10 cm	SOFT 10-16 cm
**Archaea**	untreated 6-16 cm	23			
	untreated 6-10 cm		21	59	18
	untreated 10-16 cm			18	60
	SOFT 6-10 cm				19

Stagars et al.

Table S3: Percentage of shared bacterial and archaeal 16S rRNA $OTU_{0.945}$.

		No. of samples	Max shared $OTU_{0.945}$ (%)*	Mean shared $OTU_{0.945}$ (%)*	Min shared $OTU_{0.945}$ (%)*
Bacteria	untreated	6	97	56	32
	SOFT	5	96	54	31
	0 – 4 cm	3	75	71	70
	4 – 8 cm	4	75	63	54
	10 – 16 cm	4	75	67	57
Archaea	untreated	5	83	58	39
	SOFT	5	80	60	38
	6 – 10 cm	5	79	61	41
	10 – 16 cm	5	74	65	45

* Pairwise comparison of community similarity within groups of samples based on presence-absence $OTU_{0.945}$ of standardized data (resampling without replacements of 2730 sequences for bacterial 16S rRNA and 3908 sequences for archaeal 16S rRNA). Values refer to maximum, mean and minimum shared $OTU_{0.945}$ between any given pair of samples from the respective group.

Table S4. Relative abundance of 16S rRNA gene sequences retrieved from Caspian Sea untreated and SOFT sediments. Only sequences classified as *Desulfobacterales* by the SILVA NGS pipeline (release 119.1) were considered for further detailed phylogenetic analysis using arb.

	Depth [cm]	Total *Desulfo-bacterales*	SCA1	C2-C4 alkane degr.*	SEEP1a	SEEP1b	SEEP1d	*Desulfo-sarcina*	*Desulfo-coccus* Hxd3	LCA1	LCA2	SB-29 relatives	s2551	Cyhx$	*Desulfo-bacula*	*Desulfati-glans* group	Sva0081
Untreated	0-1	14,0	0,6	0,1	0,1	0,3	1,0	0,9	0,2	0,1	0,2	0,4	0,7	0,2	2,3	0,9	2,5
	2-4	8,3	0,3	<0.1	0,2	0,2	0,8	0,4	0,2	0,1	0,1	0,3	0,4	0,1	0,9	0,9	2,3
	4-6	12,8	0,6	0,1	0,2	0,2	1,6	0,6	0,3	0,1	0,2	0,6	0,5	0,2	0,9	1,1	4,5
	6-8	15,5	0,7	<0.1	0,3	0,6	1,1	0,4	0,2	0,1	<0.1	0,2	0,4	0,1	1,7	1,8	5,4
	10-12	13,1	0,6	<0.1	0,4	0,3	1,6	0,3	0,1	0,1	0,1	0,1	0,5	0,1	1,1	1,6	4,6
	14-16	19,4	0,6	0,1	0,4	0,9	3,4	0,5	0,3	0,2	0,3	0,3	0,5	0,3	2,0	1,8	5,7
SOFT	0-1	9,2	0,6	0,1	0,2	<0.1	0,1	0,5	0,1	<0.1	<0.1	0,3	0,5	0,5	2,4	0,9	1,8
	2-4	14,0	0,5	1,1	0,2	0,2	1,2	1,3	0,2	<0.1	0,8	0,4	0,5	0,6	2,7	1,3	3,1
	4-6	20,6	0,7	0,4	0,1	0,2	0,7	1,5	0,2	0,1	1,5	0,5	0,9	0,2	8,1	1,1	3,4
	6-8	22,3	0,8	0,7	0,3	0,4	2,7	0,6	0,2	0,1	1,9	0,2	0,7	0,4	6,4	2,1	4,2
	10-12	20,0	0,6	0,1	0,4	0,6	3,3	1,0	0,3	0,1	0,2	0,2	1,1	0,1	2,9	1,7	4,9
	14-16	15,9	0,8	0,1	0,2	0,5	3,4	0,5	0,3	<0.1	0,1	0,4	0,8	0,1	1,2	2,1	3,6

Table S5. Frequencies of archaeal 16S rRNA gene sequences retrieved from initial and SOFT core sediments (6 – 16 cm depth) that are affiliated with known taxonomic clades involved in the methane cycle. Total number of quality-trimmed archaeal 16S rRNA tag sequences: 25968 for untreated sediments and 128093 for SOFT sediments. Taxonomy according to ARB SILVA (release 119.

	**Untreated sediment** [% archaeal sequences]	**SOFT sediment**
ANME-2a-2b		0.015
ANME-2c		0.001
GoM-Arch87		<0.001
Methermicoccaceae		0.009
Methanosaetaceae	0.004	0.012
Methanocellaceae		0.002
Methanomicrobiaceae		0.702
Methanosarcinaceae		
Methanolobus	0.065	0.126
Methanococcoides	0.008	0.040
Methanosarcina	0.004	11.125
Methanosaeta	0.004	0.016
Others (ANME-3, *Methanohalophilus*		0.020
**SUM [% of all Archaea]**	**0.085**	**12.069**

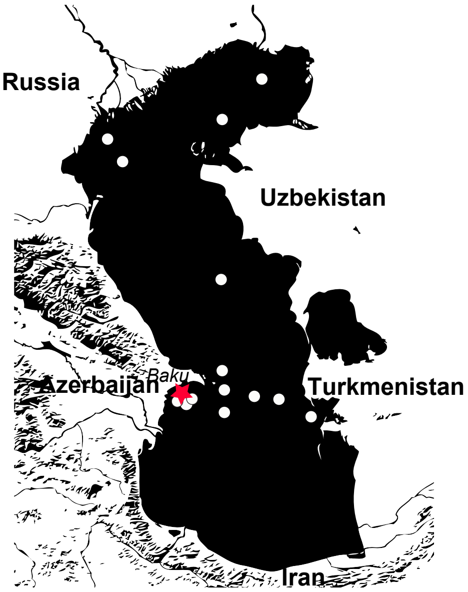

Stagars et al.

Figure S1
Map of the Caspian Sea showing major oil fields (white dots) and the sampling site off-shore Baku (Azerbaijan; red asterix).

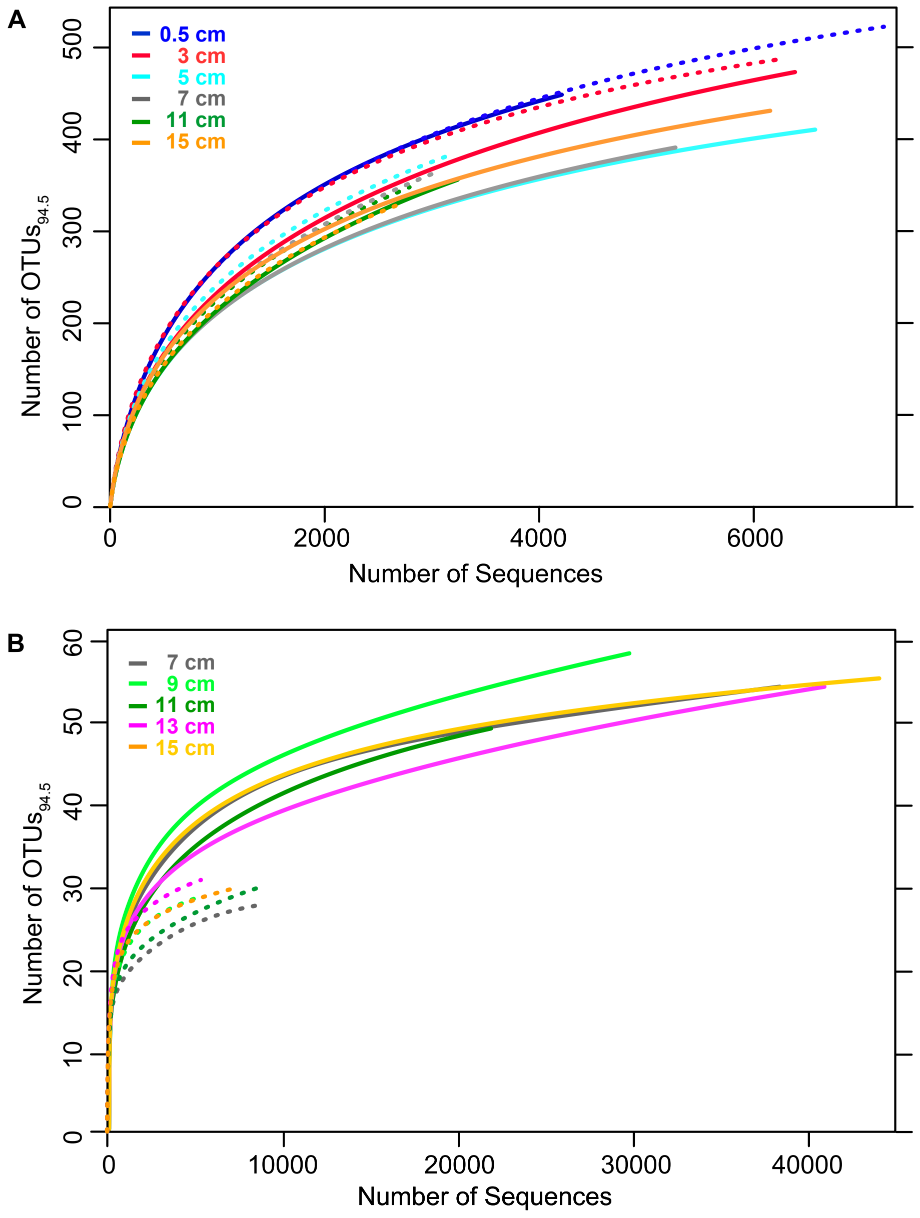

Stagars et al.

Figure S2. Rarefaction curves
Rarefaction curves for A) bacterial 16S rRNA sequences and B) archaeal 16S rRNA gene sequences clustered at 94.5% identity retrieved from SOFT (solid lines) and untreated (dashed lines) Caspian Sea sediment samples.

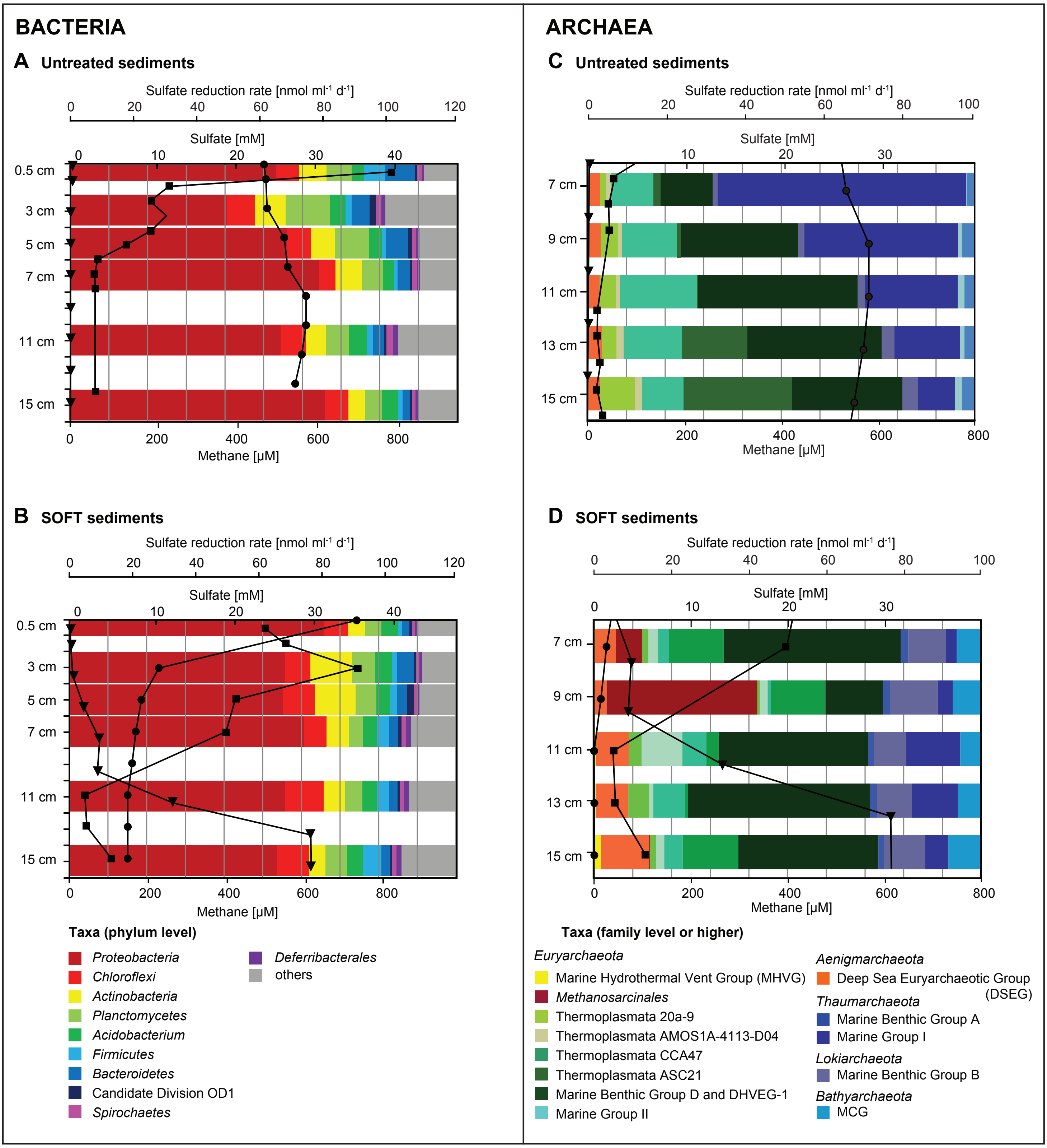

Stagars et al.

Figure S3. Microbial community composition of Caspian Sea sediments in untreated (panels A, C) and SOFT (panels B, D) sediments. Relative abundance of (A, B) bacterial taxa (based on 454-pyrosequencing of 16S rRNA genes) and (C, D) archaeal taxa (based on IonTorrent-sequencing of 16S rRNA gene) is shown. Depth profiles for methane (triangles), sulfate (dots) and sulfate reduction rates (rectangles) were taken from Mishra *et al.* (this issue).
